# Impact of Bismuth Incorporation into (Ga,Mn)As Dilute Ferromagnetic Semiconductor on Its Magnetic Properties and Magnetoresistance

**DOI:** 10.3390/ma16020788

**Published:** 2023-01-13

**Authors:** Tomasz Andrearczyk, Khrystyna Levchenko, Janusz Sadowski, Katarzyna Gas, Andrei Avdonin, Jerzy Wróbel, Tadeusz Figielski, Maciej Sawicki, Tadeusz Wosinski

**Affiliations:** 1 Institute of Physics, Polish Academy of Sciences, Aleja Lotnikow 32/46, PL-02668 Warsaw, Poland; 2 Faculty of Physics, University of Vienna, 1090 Vienna, Austria; 3 Department of Physics and Electrical Engineering, Linnaeus University, SE-391 82 Kalmar, Sweden

**Keywords:** dilute ferromagnetic semiconductors, (Ga,Mn)As, magneto-crystalline anisotropy, magnetoresistance, weak localization, spin–orbit coupling, spintronics

## Abstract

The impact of bismuth incorporation into the epitaxial layer of a (Ga,Mn)As dilute ferromagnetic semiconductor on its magnetic and electromagnetic properties is studied in very thin layers of quaternary (Ga,Mn)(Bi,As) compound grown on a GaAs substrate under a compressive misfit strain. An addition of a small atomic fraction of 1% Bi atoms, substituting As atoms in the layer, predominantly enhances the spin–orbit coupling strength in its valence band. The presence of bismuth results in a small decrease in the ferromagnetic Curie temperature and a distinct increase in the coercive fields. On the other hand, the Bi incorporation into the layer strongly enhances the magnitude of negative magnetoresistance without affecting the hole concentration in the layer. The negative magnetoresistance is interpreted in terms of the suppression of weak localization in a magnetic field. Application of the weak-localization theory for two-dimensional ferromagnets by Dugaev et al. to the experimental magnetoresistance results indicates that the decrease in spin–orbit scattering length accounts for the enhanced magnetoresistance in (Ga,Mn)(Bi,As).

## 1. Introduction

The prototype dilute ferromagnetic semiconductor (DFS) (Ga,Mn)As, where Mn atoms, substituting Ga atoms in the GaAs crystal lattice, supply magnetic moments and mobile holes responsible for p-type conductivity, has become one of the most intensively studied semiconductor materials for above two decades, e.g., [1,2,3]. Despite large progress in the optimization of epitaxial growth and post-growth annealing treatments of (Ga,Mn)As epitaxial layers, their ferromagnetic transition temperature is still much below the room temperature required for construction of functional devices. Nevertheless, this material has become especially useful for studying new concepts of spintronic devices taking advantage of electrically controlled ferromagnetism [3]. Magnetization manipulation by electric current, driven by the spin-orbit torque mechanism, is of topical interest for the next generation, energy efficient, nonvolatile memory and logic applications [4,5]. The spin-orbit torque driven magnetization switching has firstly been demonstrated on (Ga,Mn)As epitaxial layers [6] and later also on other systems containing heavy metals and strong ferromagnets, see, e.g., [7,8]. This mechanism of magnetization manipulation results from spin-orbit coupling (SOC), the phenomenon of relativistic interaction between the current carrier’s spin and its angular momentum in conducting materials. Two main effects are responsible for the appearance of SOC in solids: (1) the bulk inversion asymmetry, occurring, e.g., in GaAs crystals with a zinc-blende structure, considered by Dresselhaus [9], and (2) the structural inversion asymmetry appearing in layered crystal structures along the normal to the layer plane, considered by Bychkov and Rashba [10], known as Rashba effect. The spin–orbit interaction, in turn, strongly affects charge transport phenomena, such as magnetoresistance and anomalous Hall effect, especially in ferromagnetic conductors [11,12].

In order to increase the SOC strength in the (Ga,Mn)As DFS we have grown epitaxial layers of the quaternary (Ga,Mn)(Bi,As) compound containing a small fraction of heavy bismuth atoms, substituting arsenic atoms in (Ga,Mn)As [13,14]. As it was shown earlier, an addition of Bi into GaAs gives rise to a relativistic correction to its valence band structure and strongly enhances the spin–orbit coupling in the ternary Ga(Bi,As) compound [15,16]. Our investigations of the (Ga,Mn)(Bi,As) layers with 1% Bi content, grown by means of the low-temperature molecular-beam epitaxy method, have evidenced their high structural quality [17] and high ferromagnetic homogeneity below the Curie temperature [18], similar to those of the reference layers without the Bi content. As a result of enhanced SOC strength the (Ga,Mn)(Bi,As) layers are distinguished by significantly increased magnitudes of the anisotropic magnetoresistance [17] and planar Hall effect [19]. Moreover, our very recent experiments demonstrate also that an incorporation of just 1% Bi into (Ga,Mn)As layer results in 6-fold lowering the threshold current necessary for spin-orbit torque driven magnetization reversal with respect to that in bismuth-free (Ga,Mn)As [20].

In the present study, we thoroughly examine the impact of Bi incorporation into (Ga,Mn)As layers on their magnetic and electromagnetic properties. The (Ga,Mn)As DFS layers generally exhibit a pronounced negative magnetoresistance (MR) in strong magnetic fields at temperatures below and around the Curie temperature, *T*_C_. Such negative MR at around *T*_C_ has usually been understood as the reduction of spin-disorder scattering of charge carriers caused by the ordering of localized Mn spins in an external magnetic field, a mechanism well known in ferromagnetic metals [21]. Instead, at low temperatures, when the Mn spins in (Ga,Mn)As are fully ferromagnetically ordered, the field-induced destruction of quantum interference contribution to the resistivity caused by the effect of weak localization has been proposed to account for the negative MR [11,22,23,24].

The effect of weak localization (WL) arises due to the constructive quantum interference of two partial waves corresponding to an electron travelling diffusively along a closed trajectory in opposite directions. That interference leads to the enhanced probability of backscattering, which results in a positive contribution to electrical resistivity. The negative MR appears because a magnetic flux bounded by the closed trajectory introduces a phase difference between the time-reversed interfering waves, thus quenching WL. The magnitude of WL correction to the resistivity is limited by the time of phase coherence of the two interfering waves, which is determined by the processes of inelastic and spin-flip scattering. The presence of strong spin–orbit coupling can turn the constructive interference of partial waves into the destructive one, resulting in the so-called weak antilocalization (WAL), which leads to a positive MR at low magnetic field. However, processes causing the antilocalization are generally suppressed in ferromagnetic materials by the internal magnetic field [11,22].

## 2. Materials and Methods

The investigated 10 nm thick (Ga,Mn)(Bi,As) layer, with 6% Mn and 1% Bi contents, and the reference (Ga,Mn)As layer, of the same thickness and Mn content, were grown on semi-insulating (001)-oriented GaAs substrate by the low-temperature molecular-beam epitaxy (LT-MBE) technique at the substrate temperature of 210 °C. In situ reflection high-energy electron diffraction (RHEED) has been used to verify the DFS layer thickness and Mn composition [17,25]. The layers have been subjected to a post-growth low-temperature annealing treatment, carried out in air at 180 °C for 50 h, in order to improve their transport and magnetic properties as a result of out-diffusion of charge- and magnetic moment-compensating Mn interstitials [13,14,26,27]. High-resolution X-ray diffraction structural characterization of the similarly grown and annealed layers of 50 nm thickness have shown that both the (Ga,Mn)(Bi,As) and (Ga,Mn)As ones are grown pseudomorphically on GaAs substrate under an in-plane biaxial compressive misfit strain [17]. An addition of 1% of Bi into the (Ga,Mn)As layer resulted in a distinctly larger expansion of its lattice parameter perpendicular to the layer plane and an increase in the in-plane compressive strain to about 0.46% with respect to that of 0.26% for the (Ga,Mn)As layer [17].

**Figure 1:**
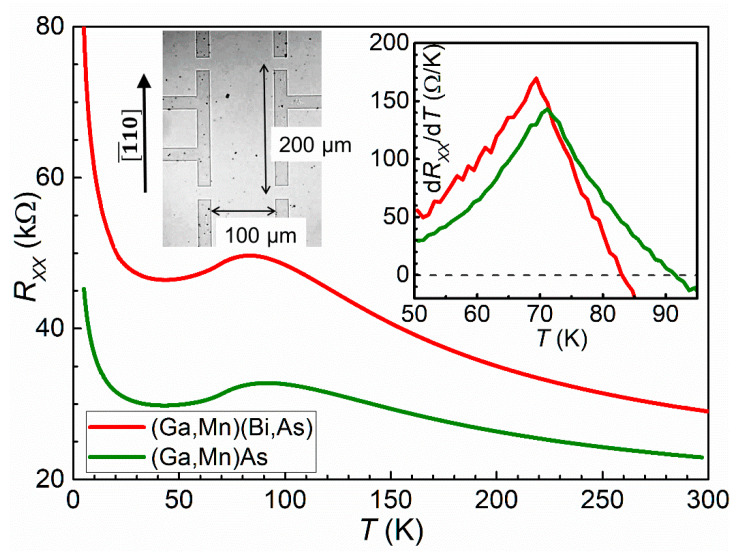
Temperature dependences of longitudinal resistance for the Hall bars of (Ga,Mn)(Bi,As) and (Ga,Mn)As layers. Right inset presents temperature derivatives of the resistance, in the temperature range around their maxima, where their intersections with zero value correspond to the maxima in the main figure. Microscopic image of the Hall bar and its geometry is shown in the left inset. Here, the darker contrast corresponds to non-conducting areas etched to the substrate.

Investigations of magnetotransport properties have been performed on Hall bars prepared from the investigated layers using electron-beam lithography patterning and chemical etching. The Hall bars of 100 μm width and 200 μm distance between the voltage contacts were aligned along the [−110] crystallographic direction of the layers. Microscopic image of the Hall-bar is shown in the left inset in Figure 1. The Hall-bars were supplied with Ohmic contacts to the (Ga,Mn)(Bi,As) and (Ga,Mn)As layers prepared by indium soldering to large contact areas located outside the image area. Four-probe longitudinal resistance *R*_xx_ and Hall resistance *R*_xy_ of the Hall-bars have been measured, using a dc ±10 μA sensing current, in a helium cryostat with superconducting electromagnet at temperatures down to 1.5 K and perpendicular magnetic field up to ±13.5 T.

Magnetic properties of the investigated layers have been studied with superconducting quantum interference device (SQUID) MPMS XL magnetometer down to 2 K for the magnetic field *H* oriented along all the main in-plane crystallographic directions and perpendicular to the layer plane. For the magnetic studies, the remaining of the In-rich metallic glue used to affix and thermalize the GaAs substrates in the MBE chamber has been removed from 5 × 5 mm^2^ specimens by means of mechanical polishing. Such a metallic contamination exerts a magnetic moment *m* of a magnitude that may well exceed that of 10 nm thin (Ga,Mn)As and exhibits a ferromagnetic-like magnetization curve *m*(*H*) [28]. On the other hand, the superconductivity of In would mar and obscure the response of (Ga,Mn)As below some 4 K, rendering the low temperature magnetic studies impossible. All the magnetic measurements have been carried out according to the well-established protocols to eliminate experimental artifacts [29].

## 3. Results and Discussion

### 3.1. Electrical Characterization

Temperature dependences of longitudinal resistance, measured at a zero magnetic field for the Hall bars of two investigated layers, are shown in Figure 1. The dependences exhibit broad maxima, characteristic of ferromagnetic materials, occurring at the vicinity of their Curie temperatures *T*_C_. They result from the spin-disorder scattering of current carriers by magnetic fluctuations while entering the paramagnetic-to-ferromagnetic phase transition [30]. These maxima at about 83 K and 92 K for the (Ga,Mn)(Bi,As) and (Ga,Mn)As layers, respectively, correspond pretty well to the *T*_C_ values determined from our SQUID magnetometry results, shown in the next section. Novák et al. [31] have shown that in (Ga,Mn)As DFS layers with rather high Curie temperatures, their values can be better estimated from maxima of temperature derivatives of resistance vs. temperature dependences, which are also shown in Figure 1 for the presently investigated layers. However, the latter maxima at about 69 K and 71 K, respectively, evidently underestimate the *T*_C_ values. Such behavior is characteristic of the (Ga,Mn)As layers with Curie temperatures of about 100 K and below [17,32]. The increase in the Hall bar resistances, observed while lowering the temperature below about 30 K, indicates that the WL correction to the Drude–Boltzmann conductivity may become dominating at low temperatures in both the investigated layers.

**Figure 2:**
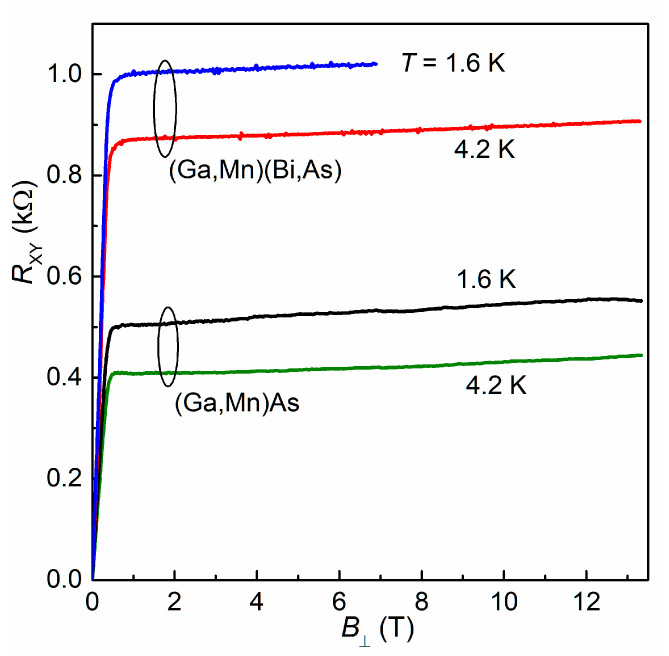
Hall resistance measured for the Hall bars of (Ga,Mn)(Bi,As) and (Ga,Mn)As layers, at temperatures of 1.6 K and 4.2 K, as a function of an external magnetic field perpendicular to the layer plane.

Hall resistance dependence on the perpendicular magnetic field *B*_⊥_ measured for the Hall bars of two investigated layers at low temperatures, 1.6 K and 4.2 K, is presented in Figure 2. For magnetic materials the Hall resistivity can be described by the relation [12]:

*ρ*_xy_ = *R*_H_*B*_⊥_ + *R*_s_*M*_⊥_, (1)

where the first term corresponds to the classical Hall effect, linear in magnetic field, which determines the type and concentration of free carriers. The second term, resulting from the spin–orbit interaction in the material, is called the anomalous Hall effect. It is proportional to the perpendicular component of the layer magnetization *M*_⊥_ and dominates at low magnetic fields. Similar hole concentrations *p* ≅ 2 × 10^20^ cm^−3^ have been determined for both the layers from the high-field (above about 1 T) results at T = 4.2 K, presented in Figure 2, where the variation of anomalous Hall effect with a magnetic field is sufficiently small.

### 3.2. Magnetic Properties

**Figure 3:**
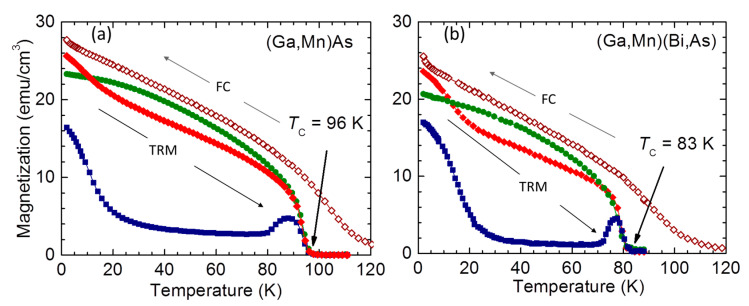
Temperature dependent magnetization *M* of (**a**) (Ga,Mn)As and (**b**) (Ga,Mn)(Bi,As) layers. On both panels open diamonds represent *M* collected during field cooling (FC) in *μ*_0_*H* = 0.1 T, with magnetic field *H* applied along the [100] in-plane direction. Solid symbols mark the thermoremnant magnetization, TRM, measured upon warming the samples in the absence of *H*, right after FC, along the in-plane crystallographic directions: [100]—red diamonds, [−110]—green bullets, and [110]—blue squares. The magnitudes of the Curie temperatures, *T*_C_, are indicated by arrows.

Results of the in-plane temperature dependent studies are summarized in Figure 3. In order to obtain a general overview of the magnetic properties, the samples are cooled down in *μ*_0_*H* = 0.1 T to the base temperature *T* = 2 K, where the field is quenched and the thermoremnant magnetization, TRM, for a given orientation is collected on warming. The warming continues until above the magnetic moment vanishes. The temperature when TRM drops to zero marks the Curie temperature, *T*_C_, for the given sample. The procedure is repeated for [100], [−110] and [110] in-plane orientations, yielding the same values of *T*_C_ = 96 K for the (Ga,Mn)As layer and *T*_C_ = 83 K for the (Ga,Bi)(Mn,As) one, regardless of the layer orientation. This visibly lower magnitude of *T*_C_ for (Ga,Bi)(Mn,As) is the first direct evidence of the influence of the enhanced SOC strength on the magnetism of (Ga,Mn)As [33].

At low temperatures, the largest TRM is observed for the [100] orientation, whereas in the mid-temperature region, the largest magnitudes of TRM are collected along the [−110] direction. The magnitudes of TRM along the [110] direction are by far smaller, yet they clearly retain non-zero values. These findings do not correspond to the general picture of the in-plane magnetic anisotropy in (Ga,Mn)As [34], in which two terms: biaxial along 〈100〉 and uniaxial along [−110] comprise the in-plane magnetic anisotropy [35,36,37,38,39]. We find that around 50 K the strongest TRM is that along [−110] orientation and it attains magnitudes nearly equal to those collected during the field cooling. This indicates that the uniaxial term is stronger than the biaxial one in a sense that the anisotropy constant of the former is larger than that of the latter. Under such circumstances, TRM measured along [110], the uniaxial hard orientation, should be negligibly small, and the magnitude of TRM measured along [100] should amount to 71% of the [−110] one [35,40]. This is not the case depicted in Figure 3. Both TRM measured along [110] and [100] exhibit larger magnitudes than expected in this two-component approach, clearly pointing to the existence of a third component to the magnetic anisotropy, a second uniaxial anisotropy with the easy axis directed along [010] [41,42].

This picture becomes even more complex at temperatures approaching *T*_C_. At about 15 K below *T*_C_, a clear hump develops on TRM [110] and TRM along [100] starts to exhibit the same magnitudes as that of [−110]. This is the signature of the beginning of the spin reorientation transition (SRT) process of the π/4 in-plane rotation of the [−110] uniaxial anisotropy [43]. This remarkable feature of (Ga,Mn)As has been observed in layers with the hole concentration exceeding *p* ≅ 6 × 10^20^ cm^−3^ and with *T*_C_ in excess of about 120 K. Since in our layers *p* ≅ 2 × 10^20^ cm^−3^ we observe only the precursory behavior of this SRT (the hump mentioned above) and its further development is hampered by relatively low magnitudes of *T*_C_. That is why instead of exchanging their intensities as observed previously in layers with much higher Curie temperatures, all three magnitudes of TRM are quenched to zero much earlier at *T*_C_. Accordingly, the quenching of TRM is sharper in (Ga,Mn)(Bi,As), which *T*_C_ is smaller than that of (Ga,Mn)As.

**Figure 4:**
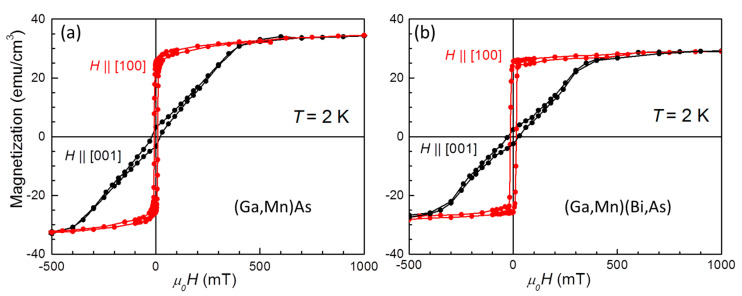
Magnetic field *H* dependence of the in-plane magnetization, represented here by the [100] one (red symbols), and of the perpendicular one (black symbols) measured at temperature *T* = 2 K for (**a**) (Ga,Mn)As and (**b**) (Ga,Mn)(Bi,As) layers studied here.

**Figure 5:**
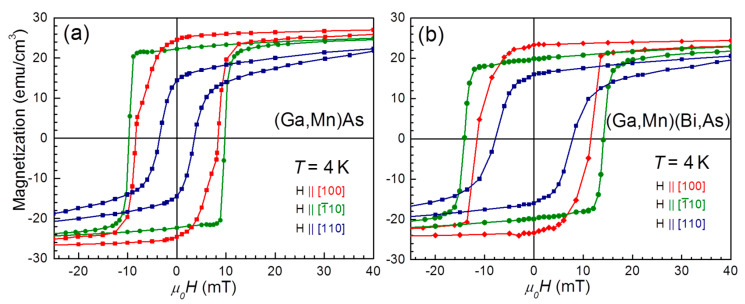
In-plane magnetic hystereses of (**a**) (Ga,Mn)As and (**b**) (Ga,Mn)(As,Bi) layers at temperature *T* = 4 K. Magnetic field *H* has been applied along three main in-plane directions, as described in the panels.

Magnetization curves *m*(*H*) for the in-plane [100] and perpendicular [001] orientations of *H* for both the layers are presented in Figure 4. These results confirm that the compressive epitaxial strain results in the easy-plane magnetization for these hole concentrations in (Ga,Mn)As [44,45], and the introduction of Bi does not affect this general picture. The corresponding perpendicular anisotropy field amounts to *μ*_0_*H_A_* ≅ 0.4 T, a value which exceeds by far the shape anisotropy in this dilute ferromagnetic material *μ*_0_*H_D_* = *μ*_0_*M_S_* ≅ 0.04 T. This discrepancy in favor of exchange effects is the direct manifestation that the magnetism in DFS, as in (Ga,Mn)As and its derivatives, is predominantly determined by the anisotropy of the carrier-mediated exchange interaction reflecting the anisotropic properties of the top of the valence band [33]. On the other hand, the magnetization hysteresis loops recorded at the magnetic field along the main in-plane crystallographic directions, shown in Figure 5, evidence the same magneto-crystalline anisotropy at low temperatures for both the investigated layers. However, the enhanced SOC strength in the Bi-contained layer results in a distinct increase in the layer coercive fields by a factor of about 1.5 for all three main in-plane crystallographic directions.

### 3.3. Magnetoresistance and Weak Localization

**Figure 6:**
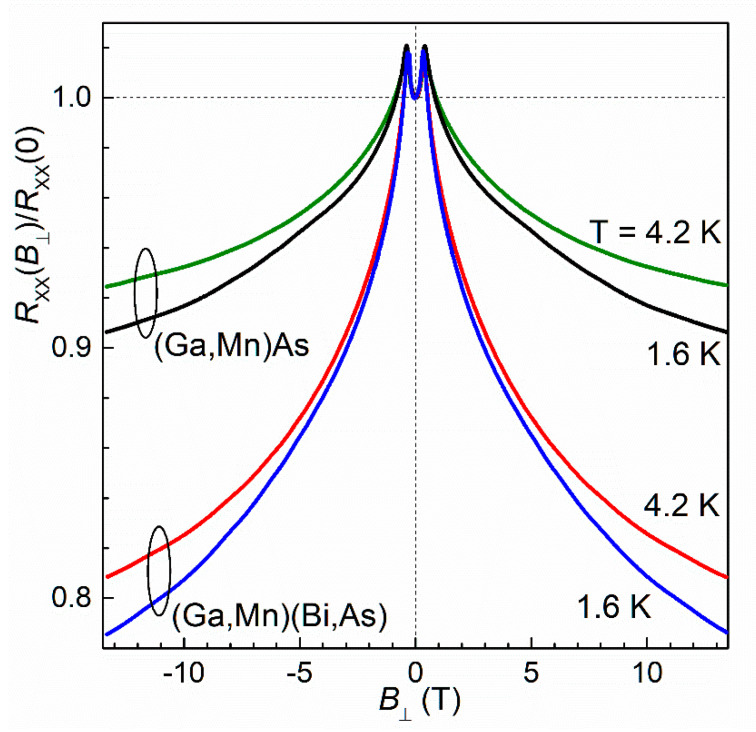
Relative longitudinal resistance measured for the Hall bars of (Ga,Mn)(Bi,As) and (Ga,Mn)As layers at temperatures of 1.6 K and 4.2 K, while sweeping an external magnetic field perpendicular to the layer plane in opposite directions.

Magnetic-field dependences of the longitudinal resistance, normalized to zero-field resistance, measured for the Hall bars of (Ga,Mn)(Bi,As) and (Ga,Mn)As layers at temperatures of 1.6 K and 4.2 K, are shown in Figure 6. At relatively weak magnetic fields, |*B*_⊥_| < 0.4 T, the dependences display a positive magnetoresistance. This positive MR is caused by the reorientation of the layer magnetization vector from its original in-plane direction at zero magnetic field to the perpendicular one at *B*_⊥_ corresponding to the perpendicular anisotropy field, which is just 0.4 T for both the studied layers, as determined from the SQUID magnetometry results shown in the previous section. We interpret this positive MR as resulting from the effect of anisotropic magnetoresistance (AMR) occurring in conducting ferromagnetic materials, which depends on the angle between the magnetization vector and the electric current direction and reaches the maximum value at the magnetization vector perpendicular to the current, cf. [17,37,46].

At higher magnetic fields, where the magnetization is saturated along the field, a negative MR, with no noticeable saturation at the highest fields, dominates. The magnitude of this negative MR increases with decreasing temperature and becomes much larger for the Bi-contained layer, reaching above 20% of the zero-field resistance for the (Ga,Mn)(Bi,As) layer at *T* = 1.6 K and *B*_⊥_ = 13 T. It reflects a significant role of both the chemical disorder and SOC that are expected to be introduced by Bi incorporation.

In order to quantitatively interpret the MR dependences, shown in Figure 6, we adapt the theory of WL developed for two-dimensional (2D) ferromagnets in perpendicular magnetic field by Dugaev et al. [22]. We apply the following expression for the quantum correction to conductivity, which takes into account the spin–orbit interaction manifesting itself as spin–orbit scattering:

(2) $$\Delta \sigma_{xx}\left(B_{⊥}\right)=-\frac{e^{2}}{2\pi^{2}\hbar }\left[\Psi \left(\frac{1}{2}+\frac{B_{p}+B_{so}}{\left|B_{⊥}\right|}\right)-\Psi \left(\frac{1}{2}+\frac{B_{\varphi }+2B_{so}}{\left|B_{⊥}\right|}\right)\right]$$

where *Ψ*(*x*) is the digamma function, and parameters *B_i_* are defined (assuming they are independent of the spin sense) by the scattering times *τ_i_* as *B_i_* = *ħ*/(4*eDτ_i_*), where *D* is the diffusion coefficient, and the index *i* stands for the following scattering processes: *p*—elastic, *φ*—inelastic, *so*—spin–orbit. The reason for applying the 2D correction for the investigated here 3D thin layers is that the phase coherence length, *L_φ_* = (*Dτ_φ_*)^1/2^, at low temperatures is expected to be of the order of 100 nm [47,48], which is much larger than the layer thickness, *d* = 10 nm. Additionally, the magnetic length, *L_B_* = (*ħ*/(*eB*))^1/2^, while comparing with *d*, implies the dimensional crossover from 3D to 2D quantum correction case, as *L_B_* > *d* for *B* < 6.6 T, but even at the maximum field used, 13.5 T, *L_B_* is still of the order of *d*—about 7 nm. Taking into account Equation (2), we examine the following formula to fit the total 3D resistivity in a perpendicular magnetic field:

(3) $$\rho_{xx}\left(B_{⊥}\right)={\left(\frac{1}{\rho_{c}}+F_{\sigma }\cdot \frac{\Delta \sigma_{xx}\left(B_{⊥}\right)}{d}\right)}^{-1}$$

where *ρ_c_* is the semi-classical Boltzmann resistivity, and *F_σ_* is the scaling factor introduced by us in order to adapt the 2D WL correction to the 3D samples. Equation (3) is derived from the inversion of conductivity tensor for weak fields approximation, i.e., *µB*_⊥_ << 1 (*µ* denotes mobility of free charge carriers—holes in our case), which is fulfilled in the whole range of fields used. 

**Figure 7:**
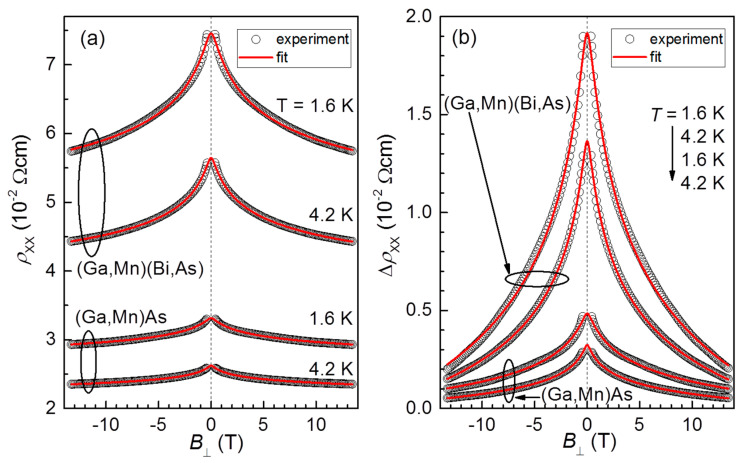
Longitudinal resistivity vs. perpendicular magnetic field measured for the Hall bars of (Ga,Mn)(Bi,As) and (Ga,Mn)As layers at temperatures of 1.6 K and 4.2 K (open circles), compared with fitting curves (red lines) within the weak localization theory for the 2D ferromagnet (see text for details). The visible set of experimental points, that are chosen to fit, is limited to the |*B*_⊥_| > 0.4 T range. The results are shown in the absolute resistivity scale (**a**) and their changes with respect to their values at the maximum field, Δ*ρ*_xx_, (vertically offset for clarity) (**b**) to compare the changes.

Figure 7 presents a comparison of experimental and fitted MR dependences for (Ga,Mn)(Bi,As) and (Ga,Mn)As layers. Although the fitting Equation (3) has been used to fit the experimental results in the field range |*B*_⊥_| > 0.4 T, where the negative MR appears, the fitted curves in Figure 7 are plotted in the whole range of fields to show that the theory accounts for the negative MR around *B*_⊥_ = 0 as well. The fitting procedure includes three fitting parameters: *ρ_c_*, *F_σ_*, and the spin–orbit scattering length *L_so_* = (*Dτ_so_*)^1/2^. Further, there are four fixed-value parameters: the technological layer thickness *d* = 10 nm, reduced effective mass of holes, set to be 0.7 [23], the hole concentration, *p*, set to be 2 × 10^20^ cm^−3^, determined from the Hall effect results, and *L_φ_*(*T*). We set *L_φ_* as fixed, because when fitting both *L_φ_* and *L_so_*, the fitting procedure is not convergent. We set *L_φ_*(*T* = 1.6 K) = 100 nm based on our previous estimation for (Ga,Mn)As nanoconstriction [47], and consequently *L_φ_*(*T* = 4.2 K) is taken equal to 50 nm according to the *L_φ_*(*T*)~*T*^−3/4^ temperature dependence that is expected for the 3D disordered systems, cf. [49].

Table 1 summarizes the obtained fitting parameters. The presented results show that the fitted values of *ρ_c_* are lower than the corresponding experimental *ρ_xx_*(*B* = 0) ones that are due to the quantum correction Δ*σ_xx_* at the *B* = 0 limit, which takes the non-zero negative value. Note that both, Δ*σ_xx_* and *ρ_c_*, are related to each other by the momentum relaxation time *τ_p_*. The obtained *ρ_c_* values correctly reproduce the increase of *ρ_xx_*(*B* = 0) with decreasing temperature and their larger values for the Bi-contained layer. On the other hand, the fitted spin–orbit scattering length, *L_so_*, reveals distinctly lower values for the Bi-contained layer, as seen in Table 1, in agreement with the enhanced SOC strength. 

**Figure 8:**
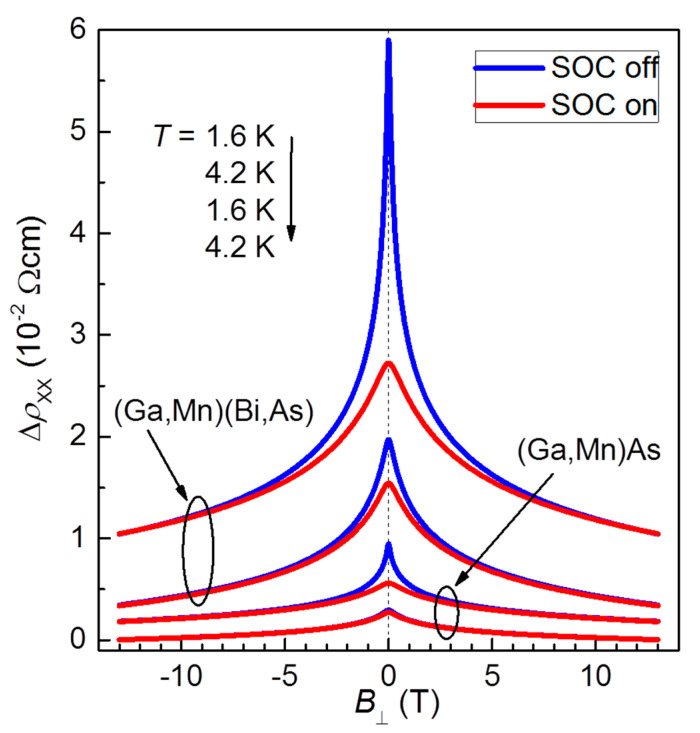
The fitted curves, the same as in Figure 7b, (red) compared with the curves calculated by Equation (3) (blue) with the spin–orbit interaction switched off, i.e., *L_so_* set as infinite (*B_so_* = 0), and with the rest of parameters unchanged comparing the red curves. The curves are vertically offset for clarity.

The role of SOC contribution to the total MR is presented in Figure 8, where the theoretical curves are compared for two cases: SOC switched on (as in Figure 7) and off (by setting *L_so_* = ∞). The visible difference between the corresponding curves implies that the spin–orbit interaction leads to antilocalization at zero magnetic field and to a positive contribution to the total MR at low field range. This positive contribution (WAL phenomenon) manifests itself more significantly for the Bi-contained layer and while lowering temperature. However, the effect is relatively weak and is dominated and obscured by the negative contribution of WL.

Table 2 lists the charge transport parameters, derived from the fitted *ρ_c_* values and measured hole concentrations in the layers. The product of the Fermi wave vector, *k_F_*, and mean free path, *l*, that is a measure of disorder, is seen to be smaller than one, indicating the charge transport regime in studied samples is actually a border between the WL and Anderson–Mott localization. We assign this conclusion as a reason for not perfect agreement between the fitted and experimental curves (compare curvatures in Figure 7), and partly for the fitted values of factor *F_σ_* (see Table 1), which are smaller than one. It turns out that fitting the experimental MR needs about 10 times smaller magnitude (*F_σ_* ~ 0.1) of WL correction than that expected by the 2D theory.

The relatively low mobility of holes (see Table 2) being of the order of 1 cm^2^/(Vs) is typical for (Ga,Mn)As layers and results from heavy doping with Mn ions. Since the values in Table 2 are determined based on the classical contribution (*ρ_c_*) to the total resistivity, thus the obtained mobility values are expected to reflect the classical momentum scattering. It is clearly seen that Bi incorporation into the layer reduces the hole mobility, confirming the increased disorder. On the other hand, the influence of low temperatures on the mobility seems to be rather weak in this classical approach.

## 4. Conclusions

The detailed investigations of the impact of Bi incorporation into (Ga,Mn)As layer, grown under compressive misfit strain, on its magnetic and electromagnetic properties have been performed by means of SQUID magnetometry and low-temperature magnetotransport measurements at high magnetic field. An addition of 1% Bi atoms, aimed at the enhancement of the spin–orbit coupling strength in this dilute ferromagnetic semiconductor, results in (i) a decrease in the ferromagnetic Curie temperature by about 15% and (ii) a distinct increase, by a factor of about 1.5, in the layer coercive fields for all three main in-plane crystallographic directions. On the other hand, the Bi incorporation into the layer (iii) does not change the hole concentration in the layer, but (iv) reduces the hole mobility by a factor of 1.5 at low temperatures, as a result of the increased chemical disorder. We also reveal (v) a strong enhancement of the magnitude of negative magnetoresistance in the (Ga,Mn)(Bi,As) layer, which is interpreted in terms of the suppression of weak localization in a magnetic field. Fitting the weak-localization theory for 2D ferromagnets to the experimental MR results confirms the enhanced spin–orbit coupling strength in the Bi-contained layer, which manifests itself in (vi) a decrease in the spin–orbit scattering length by a factor of 2, at the temperature of 4.2 K, with respect to that in the Bi-free layer.

Our extensive research provides an important contribution towards elaboration of suitable materials for spin–orbit torque driven magnetization manipulation, what is of topical interest for the next generation, energy efficient, nonvolatile memory and logic applications in sustainable society.

## Figures and Tables

**Table 1:** The best fit values of fitting parameters: *ρ_c_*, *F_σ_*, and *L_so_*, corresponding to the fitted curves in Figure 7.

Layer	*T* (K)	*ρ_c_* (10^−2^ Ωcm)	*F* *_σ_*	*L_so_* (nm)
(Ga,Mn)(Bi,As)	1.6	3.0	0.13	44
	4.2	2.5	0.15	70
(Ga,Mn)As	1.6	2.1	0.12	52
	4.2	1.8	0.11	140

**Table 2:** Hole mobilities, *µ*, and disorder parameter, *k_F_l*, derived from the fitted *ρ_c_* and measured *p* values.

Layer	*T* (K)	*µ* (cm^2^/(Vs))	*k_F_l*
(Ga,Mn)(Bi,As)	1.6	1.0	0.23
	4.2	1.2	0.27
(Ga,Mn)As	1.6	1.5	0.32
	4.2	1.7	0.37

## Data Availability

The data presented in this study are available on reasonable request from the corresponding author.
